# Case report: Melanosis coli combined with colon cancer, causality or coincidence?

**DOI:** 10.3389/fsurg.2022.973883

**Published:** 2022-08-31

**Authors:** Wei Zhao, Jianan Chen, Hongbin Xing, Jun Yu, Qian Liu

**Affiliations:** ^1^ Department of Colorectal Surgery, National Cancer Center/National Clinical Research Center for Cancer/Cancer Hospital, Chinese Academy of Medical Sciences and Peking Union Medical College, Beijing, China; ^2^ Department of General Surgery, Affiliated Cancer Hospital of Zhengzhou University, Henan Provincial Cancer Hospital, Zhengzhou, China; ^3^ Department of Surgery, the Johns Hopkins University School of Medicine, Baltimore, MD, United States

**Keywords:** melanosis coli, colonic neoplasms, colonic polyps, colectomy, case report

## Abstract

The habitual use of laxative containing anthraquinone glycosides is considered to be the main cause of melanosis coli (MC). In the past, most scholars considered MC to be a benign and reversible disease. However, new evidence has emerged that MC may increase the risk of colon cancer. Here, we report a case of a 48-year-old woman diagnosed with MC and colon cancer. Through a literature review of previous basic and clinical studies, we summarize existing evidence that reveals the possible association between MC and colon cancer. Although this case cannot establish causality between MC and colon cancer, a high level of clinical vigilance for occurrence of colon cancer in patients with MC should be maintained.

## Introduction

The habitual use of laxative containing anthraquinone glycosides is considered to be the main cause of melanosis coli (MC) (1, 2). In the past, most scholars considered MC to be a benign and reversible disease (3). In recent years, however, new evidence has emerged that MC may increase the risk of colon cancer (4). Here, we report a case of MC combined with colon cancer. Through a literature review of previous basic and clinical studies, we summarize existing evidence that reveals the possible association between MC and colon cancer.

## Case report

**Figure 1:**
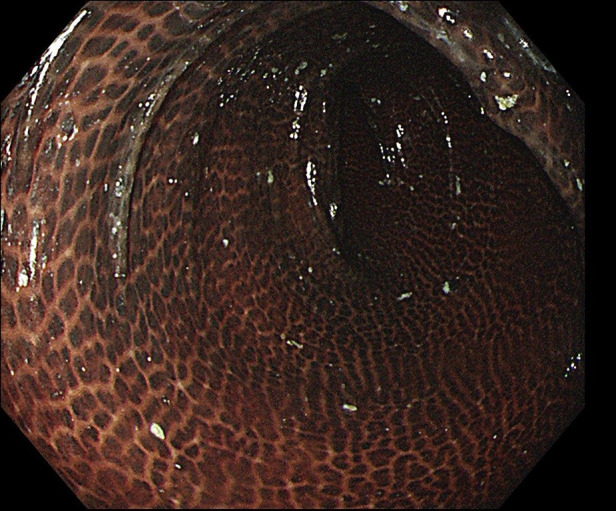
The colonoscopy revealed a colon that was diffusely filled with dark brown pigmentation in a snakeskin-like pattern.

**Figure 2:**
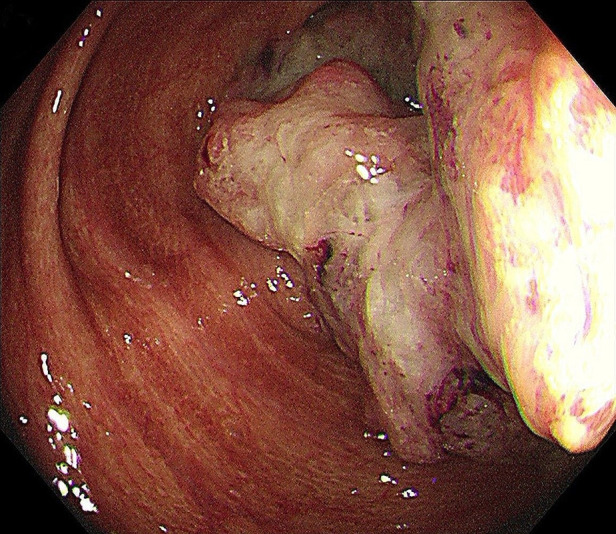
In the ileocecal region, a circumferential, irregular, ulcerative mass was found.

**Figure 3:**
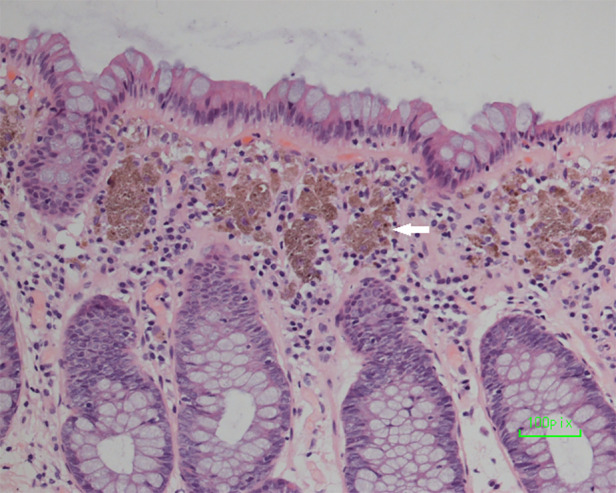
Biopsy of the dark brown intestinal mucosa showed that there were different degrees of macrophage deposition in the lamina propria interstitial cells of the colonic mucosa (white arrowhead), but the epithelial cell layer was normal.

**Figure 4:**
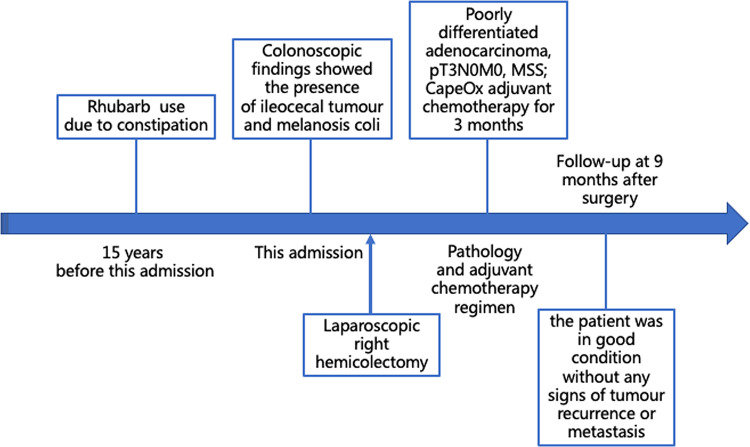
Patient's timeline of anthraquinone laxative use, colonoscopy findings, and treatment. MSS, microsatellite stability.

A 48-year-old Asian woman presented to the colorectal department with a 2-month history of abdominal pain and bloating. She had suffered from chronic constipation for the past 15 years and had taken rhubarb as needed (rhubarb is an herb, which is grown chiefly in China and can be used in folk medicine as a laxative). The patient and her first degree relatives had no history of malignant tumours. In addition, we found no clear known risk factors for colon cancer in this patient. Her colonoscopy revealed that her colon was diffusely filled with dark brown pigmentation in a snakeskin-like pattern (Figure 1). In the ileocecal region, a circumferential irregular ulcerative mass was found (Figure 2). There were also two polypoid masses located in the descending colon (Supplementary Figures S1A,B). On colonoscopy, the patient was diagnosed with an ileocaecal tumour, descending colon polyps and MC. A biopsy of the ileocecal ulcerative lesions confirmed a moderately differentiated adenocarcinoma. Biopsy of the dark brown intestinal mucosa showed that there were different degrees of macrophage deposition in the lamina propria interstitial cells of the mucosa, but the epithelial cell layer was normal (Figure 3). Laboratory studies of the patient's blood revealed a CEA level of 45.57 ng per millilitre (reference range, 0.0–5.0), a CA19-9 level of 30.53 U per millilitre (reference range, 0.0–27.0) and a haemoglobin level of 90 g per litre (reference range, 110–150 g per litre). The diagnosis of ileocecal malignancy was clear, and a laparoscopic right hemicolectomy was performed on June 2nd, 2021. The operation time was 186 min, and the intraoperative blood loss was 20 ml. There were no intraoperative blood transfusions or intraoperative complications. The pathological diagnosis was a poorly differentiated adenocarcinoma of the ileocecal region; the pathological staging was pT3N0M0 (according to AJCC 8th TNM staging system); the histological grade of the tumour was G3; there was no vascular cancer thrombus or nerve invasion; the surgical margin was negative; the number of examined lymph nodes was 30, none of which was positive for cancer; there were no cancer nodules; immunohistochemistry revealed MLH1 (+), MSH2 (+), MSH6 (+), PMS2 (+), and BRAF (V600E) (−) of tumour cells, and the positive rate of Ki-67 expression was 40%. Genetic testing did not detect mutations in known inherited tumour genes. The patient had an uneventful recovery with no postoperative complications or secondary surgery, and the postoperative hospital stay was 7 days. Postoperative adjuvant chemotherapy with the CapeOx (capecitabine and oxaliplatin) regimen was administered for 3 months. At 3 months after surgery, a CT scan and colonoscopy revealed no signs of recurrence or metastasis, and the pigmentation of colonic mucosa was reduced. At 9 months after surgery, a telephone follow-up indicated that the patient was in good condition without any signs of tumour recurrence or metastasis, and the patient said, “After surgical treatment, my abdominal pain and abdominal distention disappeared. On the doctor's advice, I regularly ate vegetables rich in fibre and fruits. My quality of life has improved.” Figure 4 indicates the clinical course of the patient.

## Discussion

Although MC is known as a very rare condition, recent study reported a rate of 1.8% MC among approximately 350,000 cases (3). In 1830, Andral and Cruveilhier first reported melanosis of the large intestine and suggested that it was related to the deposition of lipofuscin in the lamina propria of the large intestine. It develops after a few months of taking anthraquinone laxative, including rhubarb and senna. Its pathogenesis is that anthraquinones directly damage colonic epithelial cells and induce apoptosis of colonic epithelial cells (5). After macrophages engulf the apoptotic cells, lipofuscin is produced by lysosomes, and then the lipofuscin migrates to the lamina propria of the intestinal mucosa, resulting in intestinal mucosal pigmentation (6).

Most scholars believe that MC is a benign and reversible noninflammatory intestinal mucosal lesion (3). As knowledge has progressed, it was found that patients with MC had a high adenoma detection rate, which may be related to the deep pigmented background of the intestinal mucosa in MC patients because this pigment increases the visibility of adenomas (7). However, there is ongoing debate about whether MC increases the risk of colon cancer (8–11). Van Gorkon et al. (12) found that anthraquinone laxative can enhance cell proliferation activity, inhibit cell apoptosis, and may promote cell carcinogenesis. The European Food Safety Authority has reviewed the published scientific evidence on a possible link between the use of anthraquinone laxative and colon cancer and has confirmed that anthraquinone laxative should be considered genotoxic and carcinogenic (13). Some scholars have analysed the proteomic differences between colon melanosis tissues, colon cancer tissues, and normal colon mucosa by proteomic techniques, and some proteins related to colon cancer have been found in MC tissues (14, 15). In addition, prospective studies have shown a potential association between MC and cancer (16). It is therefore possible that a link may exist between MC and colon cancer. However, by far, there is still lack of convincing evidence to indicate that MC is related to colon cancer and further studies are required to clarify this point.

In our case, it is still unclear as to whether colon cancer is correlated with MC, or developed simply by coincidence. Although more cases and studies are required to support a causal relationship, it may be prudent to consider the alternative explanation that the patient coincidentally developed colon cancer of unknown cause and MC within 15 years of anthraquinone laxative use. This case should alert clinicians to possibility of colon cancer after the initial diagnosis of MC. For patients diagnosed with MC, scheduling regular clinical review should also be recommended. In addition, we recommended that anthraquinone laxatives be used only for treatment rather than for the prevention of constipation. Regular intake of vegetables rich in fibre and fruits can be considered for prevention on a regular, long-term basis. It is worth noting that the patient in this case anthraquinone laxative because of chronic constipation. However, some studies demonstrated no increase in prevalence of colorectal cancer in patients or individuals with constipation (17).

Although this case cannot establish causality between MC and colon cancer, a high level of clinical vigilance for occurrence of colon cancer in patients with MC should be maintained. More basic and clinical studies are needed to further explore the relationship between MC and colon cancer.

## Data Availability

The raw data supporting the conclusions of this article will be made available by the authors, without undue reservation.

## References

[1] Cao Y He Y Wei C Li J Qu L Zhang H Aquaporins alteration profiles revealed different actions of senna, sennosides, and sennoside a in diarrhea-rats. Int J Mol Sci. (2018) 19(10):3210. 10.3390/ijms19103210 PMC6213963 30336596

[2] Nusko G Schneider B Schneider I Wittekind C Hahn EG. Anthranoid laxative use is not a risk factor for colorectal neoplasia: results of a prospective case control study. Gut. (2000) 46(5):651–5. 10.1136/gut.46.5.651 10764708 PMC1727932

[3] Wang S Wang Z Peng L Zhang X Li J Yang Y Gender, age, and concomitant diseases of melanosis coli in China: a multicenter study of 6,090 cases. PeerJ. (2018) 6:e4483. 10.7717/peerj.4483 29568709 PMC5845562

[4] Lombardi N Crescioli G Maggini V Bellezza R Landi I Bettiol A Anthraquinone laxative use and colorectal cancer: a systematic review and meta-analysis of observational studies. Phytother Res. (2022) 36(3):1093–102. 10.1002/ptr.7373 35040201 PMC9305424

[5] Cheng Y Zhang H Qu L He Y Routledge MN Yun Gong Y Identification of rhein as the metabolite responsible for toxicity of rhubarb anthraquinones. Food Chem. (2020) 331:127363. 10.1016/j.foodchem.2020.127363 32590269

[6] Byers RJ Marsh P Parkinson D Haboubi NY. Melanosis coli is associated with an increase in colonic epithelial apoptosis and not with laxative use. Histopathology. (1997) 30(2):160–4. 10.1046/j.1365-2559.1997.d01-574.x 9067741

[7] Blackett JW Rosenberg R Mahadev S Green PHR Lebwohl B. Adenoma detection is increased in the setting of melanosis coli. J Clin Gastroenterol. (2018) 52(4):313–8. 10.1097/MCG.0000000000000756 27820223

[8] Biernacka-Wawrzonek D Stepka M Tomaszewska A Ehrmann-Josko A Chojnowska N Zemlak M Melanosis coli in patients with colon cancer. Prz Gastroenterol. (2017) 12(1):22–7. 10.5114/pg.2016.64844 28337232 PMC5360664

[9] Cowley K Jennings HW Passarella M. Who turned out the lights? An impressive case of melanosis coli. ACG Case Rep J. (2015) 3(1):13–4. 10.14309/crj.2015.85 26504866 PMC4612746

[10] Hung CY Shyung LR Chen MJ. Pigmentation sparing on melanosis coli. Gastroenterology. (2012) 142(3):e10–1. 10.1053/j.gastro.2011.07.032 22285363

[11] Kassim SA Abbas M Tang W Wu S Meng Q Zhang C Retrospective study on melanosis coli as risk factor of colorectal neoplasm: a 3-year colonoscopic finding in Zhuhai Hospital, China. Int J Colorectal Dis. (2020) 35(2):213–22. 10.1007/s00384-019-03435-7 31823053

[12] van Gorkom BA Karrenbeld A van Der Sluis T Koudstaal J de Vries EG Kleibeuker JH. Influence of a highly purified senna extract on colonic epithelium. Digestion. (2000) 61(2):113–20. 10.1159/000007743 10705175

[13] EFSA Panel on Food Additives and Nutrient Sources added to Food (ANS), Younes M Aggett P Aguilar F Crebelli R Filipic M Frutos M.J Safety of hydroxyanthracene derivatives for use in food. EFSA J. (2018) 16(1):e05090. 10.2903/j.efsa.2018.5090 32625659 PMC7009633

[14] Yuan S Wang P Zhou X Xu J Lu S Chen Y Differential proteomics mass spectrometry of melanosis coli. Am J Transl Res. (2020) 12(7):3133–48. 32774690 PMC7407713

[15] Zhou X Wang P Zhang YJ Xu JJ Zhang LM Zhu L Comparative proteomic analysis of melanosis coli with colon cancer. Oncol Rep. (2016) 36(6):3700–6. 10.3892/or.2016.5178 27748902

[16] Siegers CP Hertzberg-Lottin von E Otte M Schneider B. Anthranoid laxative abuse-a risk for colorectal cancer? Gut. (1993) 34(8):1099–101. 10.1136/gut.34.8.1099 8174962 PMC1374362

[17] Power AM Nicholas TJ Alexander FC. Association between constipation and colorectal cancer: systematic review and meta-analysis of observational studies. Am J Gastroenterol. (2013) 108(6):894–903. 10.1038/ajg.2013.52 23481143

