# Supplementary figures and images for: Case report: Melanosis coli combined with colon cancer, causality or coincidence?
Source: Front Surg. 2022 Aug 31;9:973883. doi: 10.3389/fsurg.2022.973883 (PMC9471420; doi:10.3389/fsurg.2022.973883)

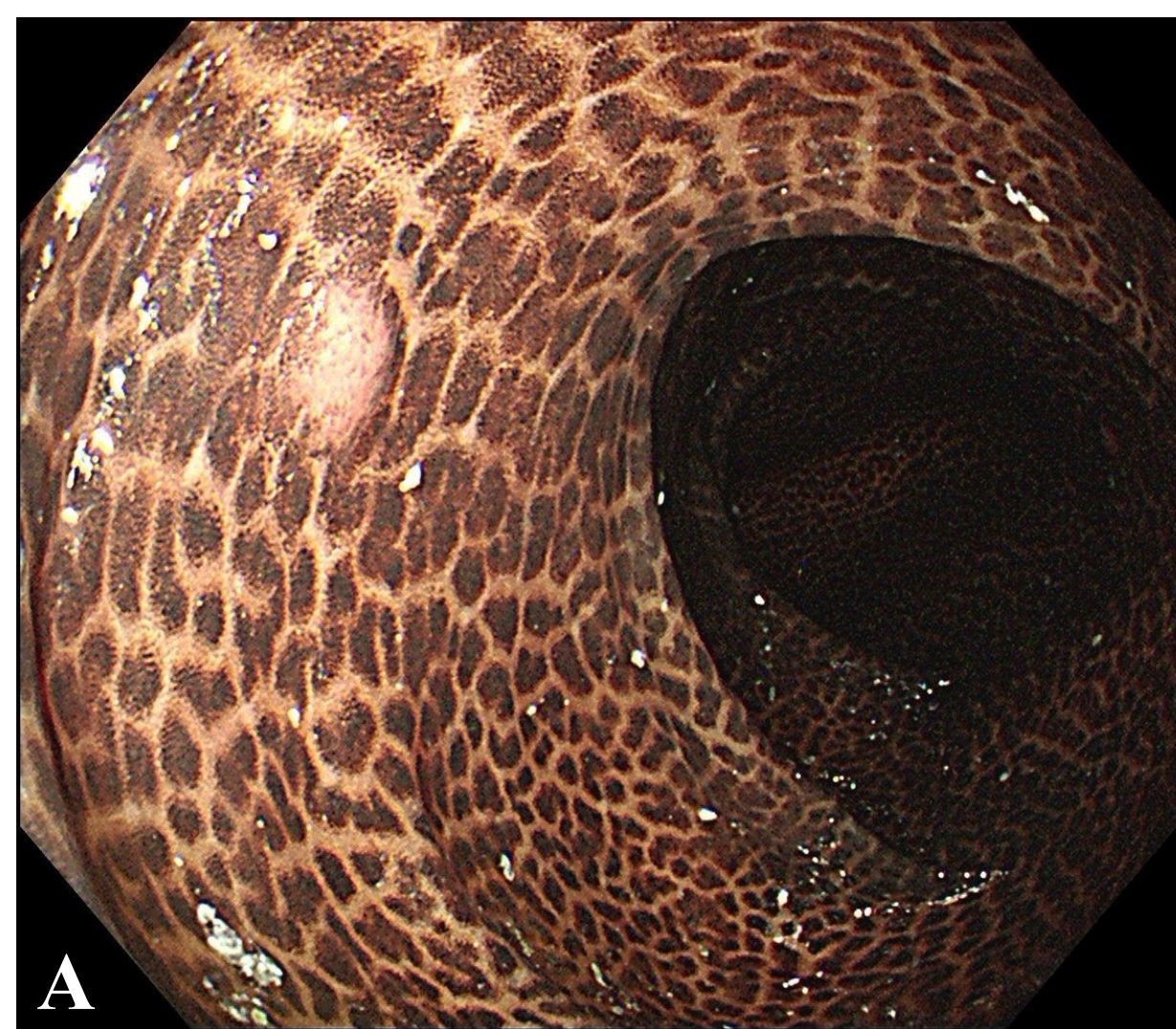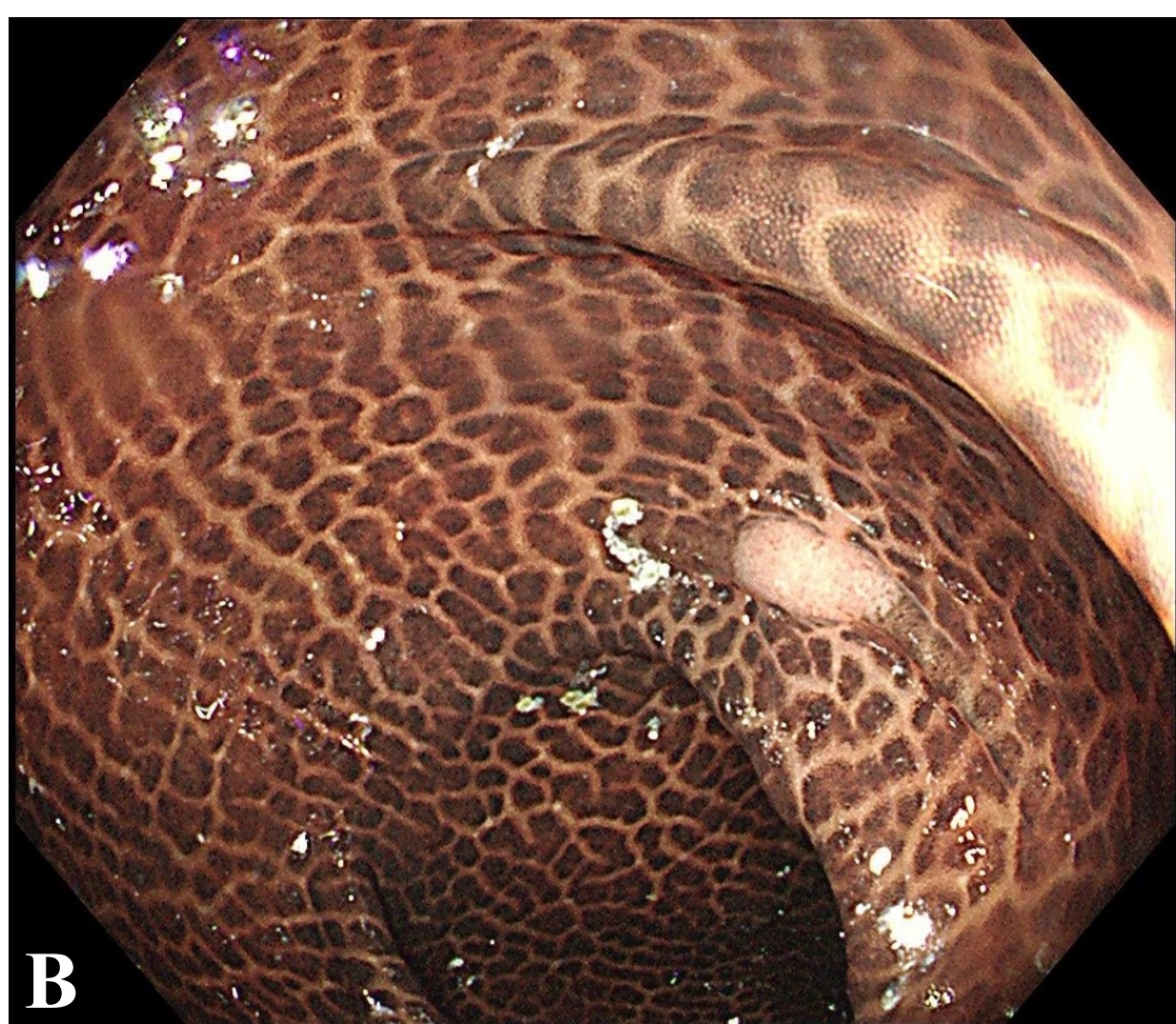

Supplement: Supplementary file 1 [file Data_Sheet_1_v1.pdf]
